# Vulvar Fibroadenoma with Lactational Changes in Ectopic Breast Tissue

**DOI:** 10.1155/2013/924902

**Published:** 2013-10-09

**Authors:** Naama Lev-Cohain, Payal Kapur, Ivan Pedrosa

**Affiliations:** ^1^Department of Radiology, University of Texas Southwestern, 2201 Inwood Road Second Floor, Dallas, TX 75390-9085, USA; ^2^Department of Pathology, University of Texas Southwestern, 5323 Harry Hines Boulevard Dallas, TX 75390-9072, USA

## Abstract

Ectopic breast tissue represents any type of breast tissue found outside its normal location in the pectoral region. The second most common location for ectopic breast tissue after axilla is the vulvar region. We present a case of a healthy 20-year-old female, G1P1, who presented to the Emergency Department with a sudden increase in size of a painful mass located in her vulva, which started 4 days after a spontaneous vaginal delivery and 3 days after initiation of breast-feeding of her newborn. She reported a stable, smaller, painless mass in the same location for almost 2 years prior to this episode. After surgical excision, a fibroadenoma with lactation changes within ectopic breast tissue was confirmed.

## 1. Introduction

Ectopic breast tissue can occur outside the pectoral area in virtually any location of the torso with the axillary region being the most common location [1]. The vulvar region represents the second most common location and ectopic tissue in this region is sensitive to hormonal changes, similar to glandular tissue in the breast [1]. The differential diagnosis of a mass in the vulva includes both benign and malignant neoplastic processes [2]. Magnetic resonance imaging (MRI) is increasingly used to evaluate masses in this anatomic location because its superb soft-tissue contrast and multiplanar capability provide exquisite detail prior to surgical resection. However, the MRI findings are rarely characteristic enough to allow for a specific diagnosis. We present a case where the clinical presentation however provided a critical clue for the correct diagnosis. Our patient reported a chronic painless mass in the vulva, which increased in size rapidly and became painful coincidentally with the beginning of breast-feeding of her newborn, indicating a possible hormonal effect [3]. In this report we discuss the differential diagnosis for masses in the vulvar area and the MRI findings that may suggest specific benign and malignant diagnoses. The importance of the clinical presentation for the elaboration of a differential diagnosis is emphasized.

## 2. History

A previously healthy 20-year-old female, G1P1, with no previous medical history presented to the Emergency Department 6 days after a spontaneous vaginal delivery of a full-term healthy male neonate. Her main complaint was sudden increase in size of a painful mass in her right vulvar labia, which started 4 days after delivery. She reported a stable, painless mass in the same location for almost 2 years, for which she did not undergo further workup. Physical examination found a rounded, very tender, right labial mass and low-grade fever. The patient underwent MRI of the pelvis.

## 3. Imaging Findings

**Figure 1:**
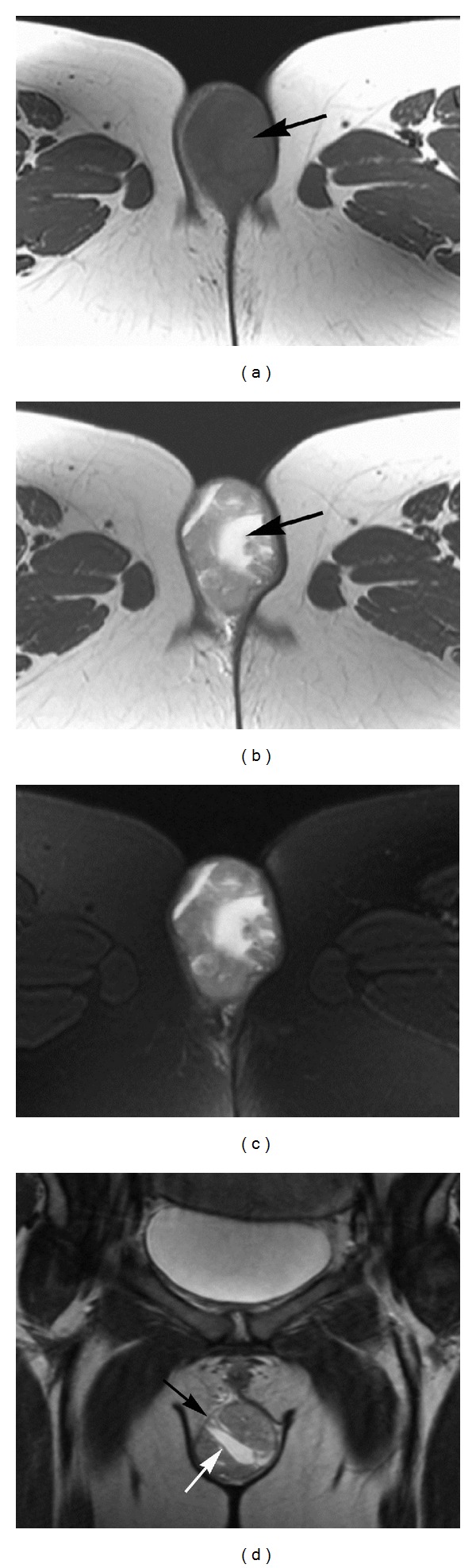
Magnetic resonance images of the pelvis. (a) Axial T1-weighted spin-echo image (TR = 700 ms/TE = 10 ms). A well-defined, ovoid mass in the subcutaneous tissue of the inferior right labia majora is seen (arrow), with smooth contours and no evidence of infiltration of adjacent structures. The mass has homogenous signal which is isointense to skeletal muscle. (b) Axial T2-weighted fast spin-echo image (TR = 4450 ms/TE = 89 ms). The mass has heterogeneous high signal intensity, with areas of very high signal intensity suggesting the presence of fluid (arrow) (c) Axial T2-weighted fast spin-echo image with frequency selective fat saturation (TR = 5266 ms/TE = 89 ms). No inflammatory changes are seen in the adjacent fat. (d) Coronal T2-weighted fast spin-echo image (TR = 5216 ms/TE = 83 ms). A well-defined thin capsule around the mass (black arrow) and thin internal septa (white arrow) are demonstrated.

A pelvic MRI was performed on a 3T clinical system (Signa HDxt; General Electric Healthcare, Waukesha, WI) using the following sequences: T2-weighted fast spin-echo (FSE) in the axial (TR = 4450 ms, TE = 89 ms, matrix = 512 × 512, thickness = 6 mm, bandwidth = 31.2 kHz), sagittal (TR = 4900 ms, TE = 82 ms, matrix = 512 × 512, thickness = 6 mm, bandwidth = 31.2 kHz) and coronal (TR = 5216 ms, TE = 83 ms, matrix = 512 × 512, thickness = 5 mm, bandwidth = 31.2 kHz) planes; axial T1-weighted FSE (TR = 700 ms, TE = 10 ms, matrix = 320 × 192, slice thickness = 6 mm, bandwidth = 31.2 kHz); and axial T2-weighted FSE with fat saturation (TR = 5266 ms, TE = 89 ms, matrix = 512 × 512, slice thickness = 6 mm, bandwidth = 31.2 kHz). No contrast material was administered. The MRI demonstrated a 3.7 × 6.7 × 4.5 cm well-defined, ovoid mass in the subcutaneous tissue of the inferior right labia majora with smooth contours and no evidence of infiltration of adjacent structures. The mass was homogenous and isointense to skeletal muscle on T1-weighted images (Figure 1(a)) and had heterogeneous high signal intensity on T2-weighted images, with areas of very high signal intensity suggesting the presence of fluid (Figures 1(b)-1(c)). A well-defined thin capsule around the mass and thin internal septa were also demonstrated (Figure 1(d)). There was no evidence of inflammatory changes in the adjacent fat on T2-weighted fat saturated images (Figure 1(d)). Imaging findings were worrisome for a neoplastic process, and surgical excision was recommended.

**Figure 2:**
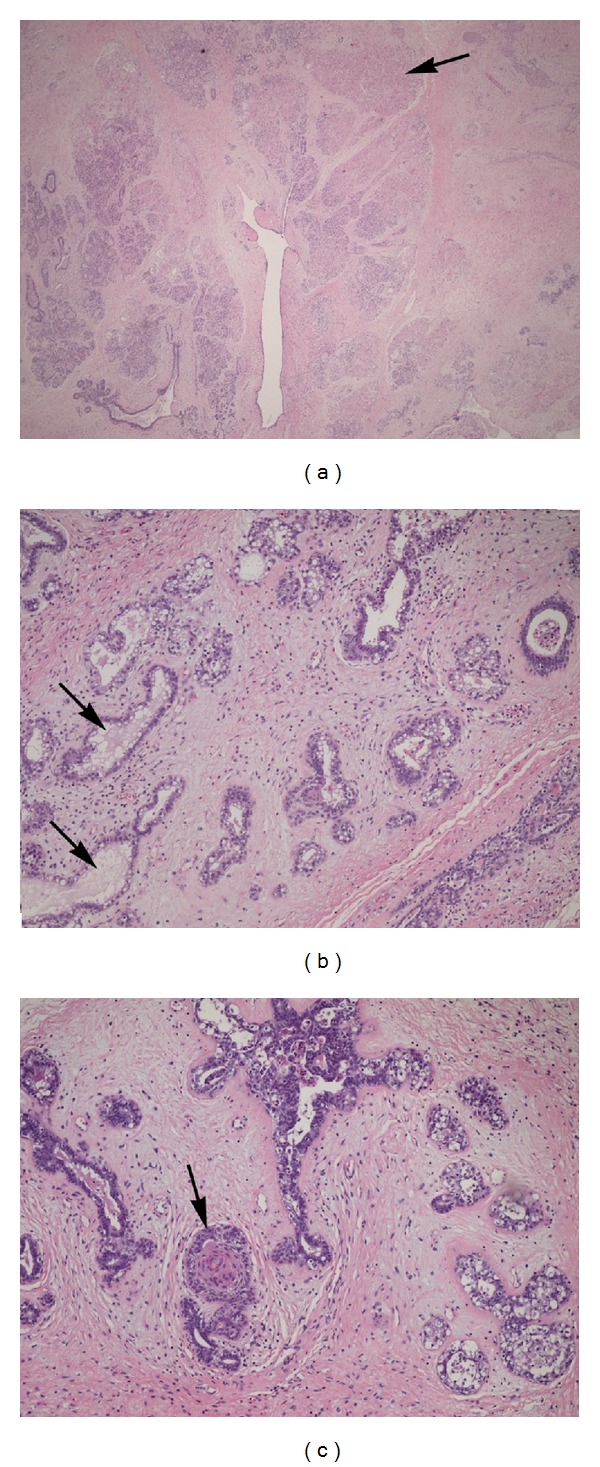
Histology slides of the lesion show (a) lobular architecture and associated ischemic necrosis (arrow). (b) Epithelial and mesenchymal proliferation with apocrine change (arrow). (c) Focal squamous metaplasia (arrow). Images were acquired using H&E staining with 40x (a) and 100x ((b), (c)) magnification.

At surgery a 7 cm × 5 cm × 4 cm well circumscribed, oval mass was removed. On gross examination, the external surface was smooth and glistening. The cut surface revealed a bulging, uniformly solid, fleshy, gray-white-to-tan, lobulated mass. Hematoxylin and eosin stain revealed the characteristic epithelial and mesenchymal proliferation seen in breast fibroadenoma (Figures 2(a) and 2(b)). Proliferation of ducts and tubules was enclosed by delicate cellular stroma that compressed or distorted the glandular components. The ducts and tubules were lined by two cell layers: a luminal epithelial cell layer and an underlying layer of myoepithelial cells. Focal squamous metaplasia (Figure 2(c)) and apocrine epithelial differentiation (Figure 2(b)) were also observed. The mass had a well-developed capsule. Focal areas of necrosis were seen (arrow Figure 2(a)). No epithelial or stromal atypia was identified. These findings were consistent with a diagnosis of fibroadenoma with lactation changes within ectopic breast tissue.

## 4. Discussion

The differential diagnosis of a mass in the vulva includes both benign and malignant neoplastic processes. Most common benign masses in this region would include cystic lesions such as Bartholin cysts, lymphangiomas, and hidradenoma papilliferum [2]. These have a characteristic MRI appearance based on their predominantly cystic nature, which was not consistent with the lesion presented in our case. Among benign solid masses, lipomas may be encountered in the vulvar region and the diagnosis is straight-forward when fatty tissue is detected by MRI. 

Squamous cell carcinoma is the most common malignant mass arising in the vulva [2]. Other malignant masses arising in this anatomic location include melanoma, basal cell carcinoma, pagets disease, sarcoma, and adenocarcinoma originating from Bartholin glands, as well as metastatic disease. Most malignant neoplasms share similar MRI characteristics with moderate high signal intensity on T2-weighted images relative to subcutaneous tissues. 

While the MRI characteristics of the mass in our case are not specific, the clinical presentation is an important consideration when elaborating a differential diagnosis. The patient reported a stable, nontender, palpable mass for 2 years that became painful and substantially larger suddenly only 4 days after delivery of her child. The rapid change in size in this clinical setting indicates a possible hormonal effect. Di Gilio et al. [3] reported a case of a myxoid vulvar leiomyosarcoma with rapid growth during pregnancy attributed to the elevated progesterone-estrogen levels. Other hormonally-sensitive lesions include lactation adenomas originating in the breast during the puerperium due to a rapid increase in the number and size of the alveoli; these regress spontaneously after ceasing lactation [4].

Ectopic breast tissue is referred to any type of breast tissue found outside its normal location in the pectoral region. The most common locations for ectopic breast tissue are the axilla, pectoral region, and vulva [1]. It has been proposed that ectopic breast tissue may originate from persistent mammary streaks which develop early in the embryonic trunk and that extend from the axilla to the groin bilaterally [5]. Alternatively, ectopic breast tissue may represent “sweat glands” commonly found in the anogenital area with a histological appearance mimicking that of mammary glands [6].

During pregnancy, high levels of estrogen, progesterone, and prolactin promote the growth and proliferation of the breast tissue and have similar effects on ectopic breast tissue. After delivery, elevated levels of prolactin and withdrawal of estrogens and progesterone result in the onset of milk secretion (lactogenesis). High levels of prolactin decline approximately 3-4 days after delivery, unless the production of prolactin remains stimulated by neonatal sucking. Our patient initiated breast-feeding on the first day after delivery, so the rapid change in size of the mass afterward is suggestive of prolactin-induced growth.

The most important differential diagnosis for an enlarging mass during pregnancy or lactation is breast cancer. However, the growth of benign lesions such as adenomas and fibroadenomas during lactation is well documented [5]. MRI changes associated with lactation include increased signal intensity in the breast parenchyma on T2-weighted images and increased vascularity, manifested by avid enhancement after administration of contrast [7]. The later was not shown in our patient because she did not receive intravenous contrast material. 

Fibroadenomas in lactating breast are often undistinguishable histopathologically from lactating adenomas. Despite being usually asymptomatic, pain may occur due to rapid growth of the tissue, in a fashion similar to that of normal situated breast tissue during pregnancy and lactation [5]. 

Most fibroadenomas show smooth margins and a round or lobulated shape, are hypo- or isointense compared with adjacent breast tissue on T1-weighted images, and variable in signal intensity on T2 weighted images [8]. They can demonstrate internal septations, which are best seen on T2-weighted images in 40% of the cases [8]. Changes during lactation include variable diameter of the tubules, apocrine secretion, and intracytoplasmic vacuoles that can be present together with cystic dilatations of the tubules inside the glandular structures [9]. To our knowledge, only 13 cases of vulvar fibroadenoma have been previously reported [10].

In summary, the vulvar region is one of the common locations for ectopic breast tissue in the body. The presence of a rapidly enlarging, well encapsulated mass in the vulvar region with heterogeneous high signal intensity on T2-weighted images associated to recent delivery and breast feeding is suggestive of lactating fibroadenoma within ectopic breast tissue. Although the MRI appearance of lactating fibroadenoma has not been reported to our knowledge, the pathologic findings were consistent with this diagnosis.
